# Taurine Supplementation Reduces Blood Pressure and Prevents Endothelial Dysfunction and Oxidative Stress in Post-Weaning Protein-Restricted Rats

**DOI:** 10.1371/journal.pone.0105851

**Published:** 2014-08-29

**Authors:** Aline R. Maia, Thiago M. Batista, Jamaira A. Victorio, Stefano P. Clerici, Maria A. Delbin, Everardo M. Carneiro, Ana P. Davel

**Affiliations:** Department of Structural and Functional Biology, Institute of Biology, University of Campinas (UNICAMP), Campinas, São Paulo, Brazil; Medical University Innsbruck, Austria

## Abstract

**Introduction:**

Taurine is a sulfur-containing amino acid that exerts protective effects on vascular function and structure in several models of cardiovascular diseases through its antioxidant and anti-inflammatory properties. Early protein malnutrition reprograms the cardiovascular system and is linked to hypertension in adulthood. This study assessed the effects of taurine supplementation in vascular alterations induced by protein restriction in post-weaning rats.

**Methods and Results:**

Weaned male Wistar rats were fed normal- (12%, NP) or low-protein (6%, LP) diets for 90 days. Half of the NP and LP rats concomitantly received 2.5% taurine supplementation in the drinking water (NPT and LPT, respectively). LP rats showed elevated systolic, diastolic and mean arterial blood pressure versus NP rats; taurine supplementation partially prevented this increase. There was a reduced relaxation response to acetylcholine in isolated thoracic aortic rings from the LP group that was reversed by superoxide dismutase (SOD) or apocynin incubation. Protein expression of p47^phox^ NADPH oxidase subunit was enhanced, whereas extracellular (EC)-SOD and endothelial nitric oxide synthase phosphorylation at Ser 1177 (p-eNOS) were reduced in aortas from LP rats. Furthermore, ROS production was enhanced while acetylcholine-induced NO release was reduced in aortas from the LP group. Taurine supplementation improved the relaxation response to acetylcholine and eNOS-derived NO production, increased EC-SOD and p-eNOS protein expression, as well as reduced ROS generation and p47^phox^ expression in the aortas from LPT rats. LP rats showed an increased aortic wall/lumen ratio and taurine prevented this remodeling through a reduction in wall media thickness.

**Conclusion:**

Our data indicate a protective role of taurine supplementation on the high blood pressure, endothelial dysfunction and vascular remodeling induced by post-weaning protein restriction. The beneficial vascular effect of taurine was associated with restoration of vascular redox homeostasis and improvement of NO bioavailability.

## Introduction

The consumption of insufficient calories, protein, vitamins and/or minerals during early stages of development might compromise the regulation of cardiovascular system homeostasis. It is especially relevant when there is an association between weight reduction and catch-up growth that can predispose an individual to the development of cardio-metabolic diseases in adulthood, such as diabetes and arterial hypertension [1]–[3].

It has been demonstrated that caloric and/or protein intrauterine undernutrition induces hypertension in offspring [4]–[6]. Moreover, endothelial dysfunction seems to be a key pathophysiological event in the cardiovascular injury observed in adult offspring [4]–[7]. Intrauterine undernutrition-induced endothelial dysfunction is associated with an impairment in nitric oxide (NO) bioavailability that can be related to reduced antioxidant defense and increased superoxide anion generation, as well as increased inflammatory markers [4]–[6]. Post-weaning protein restriction also can be related to an increase in blood pressure in adulthood, and is associated with baroreflex dysfunction and increased sympathetic tone [2], [8]. However, it is still unclear whether the hypertension induced by early life protein restriction is associated with endothelial dysfunction and vascular oxidative stress in adulthood.

Taurine is a sulfur-containing semi-essential amino acid, endogenously synthesized and also provided by diet, especially fish and meat. It has been demonstrated that taurine acts as antioxidant and anti-inflammatory agent [9]–[11] in addition to its vasodilatory proprieties [12]. In endothelial cells, taurine inhibits apoptosis, inflammation and oxidative stress while increasing NO generation [13]–[15]. It was demonstrated that taurine supplementation restores insulin secretion in islets from post-weaning low-protein rodents [16]. Therefore, we hypothesized that taurine supplementation could have beneficial effect on the cardiovascular alterations induced by a low-protein diet. In the present study, we investigated the effect of taurine supplementation on blood pressure, endothelial function and vascular morphometry of post-weaning protein-restricted rats.

## Materials and Methods

This study was approved by the Ethical Committee for Animal Research (approval number: 2653-1) at the State University of Campinas (UNICAMP) and it conforms with the guidelines for ethical conduct in the care and use of animals established by the Brazilian Society of Laboratory Animal Science (SBCAL/COBEA).

### Animals and experimental groups

Experiments were carried out on 21-day-old male Wistar rats obtained from Animal Care Facility of UNICAMP maintained at 22°C, on a 12 h light/dark cycle with free access to drink and food. Animals were housed in collective cages (N = 4 per cage) and divided into four experimental groups: rats fed an isocaloric 12% protein diet without (NP) or with 2.5% of taurine (NPT) in drinking water and rats fed an isocaloric low protein diet containing 6% protein without (LP) or with 2.5% of taurine (LPT) in drinking water. Diet composition was previously described [17] and 2.5% taurine supplementation has been reported to have beneficial metabolic effect on protein-restricted rats [16]. Rats were fed the diets and received water or taurine solution for 90 days. Body weight was measured in the beginning and at the end of the experimental period.

### Hemodynamic measurements

At the end of the diet and supplementation periods, animals were anesthetized with urethane (1.2 g/kg, *i.p.*) and a polyethylene catheter (PE-50) filled with saline (0.9%) was placed in the right common carotid artery for measurements of systolic (SBP), diastolic (DBP) and mean arterial blood pressure (MAP) and heart rate (HR). The catheter was connected to a pressure transducer (MTL844 ADInstruments, Sydney, Australia) and data acquisition was performed using PowerLab 8/30 system (LabChart 7, ADInstruments).

### Vascular reactivity

Immediately after hemodynamic measurements, animals were decapitated under anesthesia and the thoracic aorta was isolated and placed in freshly prepared ice-cold Krebs-Henseleit solution, as previously described [18]. The isolated aorta was cleaned of fat and connective tissue, cut into rings (3 mm) that were mounted in a 10 mL organ chamber bath (Panlan Harvard Apparatus, Barcelona, Spain) with Krebs-Henseleit solution at 37°C, pH 7.4, and continuously gassed with 95% O_2_, 5% CO_2_ under a resting tension of 1 g. Data acquisition was performed using PowerLab 8/30 system (LabChart 7, ADInstruments).

After the equilibration period (1 h), aortic rings were exposed to 125 mM KCl to verify smooth muscle viability and maximum contractile response. Then, rings were contracted with serotonin (∼1 µM, able to reach 50% of maximal response to 125 mM KCl) and a cumulative concentration-response curve to acetylcholine (1 nM to 10 µM) was performed to assess endothelium-dependent relaxation. To evaluate the contribution of superoxide anion from NADPH oxidase, some aortic rings were incubated for 30 minutes with superoxide dismutase, a superoxide anion scavenger (150 U/mL SOD) or apocynin, a NADPH oxidase inhibitor (30 µM), before measuring the concentration-response curves to acetylcholine. The relaxation response to the NO donor sodium nitroprusside (0.1 nM to 1 µM) was also performed in pre-contracted aortic rings.

Concentration-response curves were fitted with a sigmoidal dose-response equation with variable slope, which disclosed the maximal effect (Emax) and the negative logarithm of the concentration of agonist that produces half-maximal response (pD_2_) for each aortic segment, using GraphPad Prism (GraphPad Software In, San Diego, CA, USA).

### NO production and reactive oxygen species (ROS) detection

The oxidative fluorescent dye hydroethidine (DHE; Invitrogen, Grand Island, NY, USA) was used to evaluate ROS generation [18] whereas NO production was evaluated using the NO-sensitive fluorescent dye 4,5-diaminofluorescein diacetate (DAF-2; Sigma-Aldrich, Saint Louis, MO, USA) [19]. Segment (3 mm) of the thoracic aorta was embedded in freezing medium and transverse sections (10 µm for DAF-2; 14 µm for DHE) were obtained with a cryostat (−25°C), collected on glass slides and equilibrated for 10 min in phosphate buffer (PB 0.1 mM, pH 7.4) at 37°C.

For analysis of NO, slides were incubated for 30 min at 37°C in PB containing CaCl_2_ (0.45 mM) and DAF-2 (8 µM) in a light-protected humidified chamber at 37°C. Afterwards, sections were stimulated without (basal) or with acetylcholine (1 µM) for 15 min. Some experiments were performed in the presence of L-NAME (100 µM) to evaluate the effect of NO synthase (NOS) inhibition on the acetylcholine-induced NO production. For analysis of ROS, slides were incubated with PBS containing DTPA (100 µM) and DHE (2 µM) in a light-protected humidified chamber at 37°C for 30 minutes. Then, L-NAME (1 mM) or the cell-permeable SOD mimetic MnTMPyP (25 µM) was topically applied in some aortic sections. Negative control sections received the same volume of PBS in the absence of DAF-2 or DHE.

Images were obtained with a microscope (Olympus BX60, Olympus, Center Valley, PA, USA) equipped with a rhodamine and fluorescein filters and camera (Olympus DP-72), using a 20× objective. The fluorescence was quantified using Image J software (National Institutes of Health, Bethesda, MD, USA) by calculus of the integrative density. DAF-2 results were expressed as a percentage increase in relation to basal levels; DHE fluorescence was normalized by vessel area.

### Western blot analysis

The aortic tissue was homogenized in RIPA lysing buffer (Upstate, Temecula,CA, USA) with 1 mM Na_3_VO_4_, 1 mM phenylmethylsulphonyl fluoride and protease inhibitor cocktail (2 µL/mL; Sigma-Aldrich, St. Louis, MO, USA). The tissue lysate was centrifuged (2,500 rpm; 15 min at 4°C) and the supernatant was collected. The total protein concentration was determined by BCA protein assay kit (Pierce, Rockford, IL, USA).

Proteins (50 µg) were electrophoretically (Mini-Protean II, Electrophoresis Cell, Bio-Rad, Hercules, CA, USA) separated by SDS-PAGE and subsequently transferred to polyvinylidene difluoride membranes (PDVF, GE Healthcare, Waukesha, WI, USA). Membranes were blocked for 2 h in Tris-buffered solution (10 mM Tris, 100 mM NaCl and 0.1% Tween 20) with 5% nonfat milk and then incubated overnight at 4°C with a rabbit or mouse polyclonal primary antibodies (1∶1,000) against the NADPH oxidase subunits p47^phox^ and gp91^phox^, superoxide dismutase (Cu/Zn-SOD, Mn-SOD and EC-SOD), glutathione peroxide (GPx), catalase, endothelial NOS (eNOS) and eNOS phosphorylation at Ser1177 (p-eNOS). After washing, the membranes were incubated with specific secondary antibody conjugated to horseradish peroxidase (1∶5,000; anti-mouse or anti-rabbit IgG antibody, Bio-Rad). The immunocomplexes were detected using the enhanced horseradish peroxidase-luminol chemiluminescence system (ECL, Amersham International, Piscataway, NJ, USA) and subjected to autoradiography (Hyperfilm ECL, GE Healthcare). The same membrane was used to determine α-actin expression using a monoclonal anti α-actin antibody (1∶8,000; Abcam, Cambridge-MA, USA) and α-actin content was used as an internal control for the loading. Scanning densitometry was used to quantify the immunoblot signals using Image J software.

### Aortic morphometry

Thoracic aortic segments were fixed in 4% sodium phosphate-buffered formaldehyde, processed, embedded in paraffin and cut into traversal 5 µm-thick slices that were stained with hematoxylin and eosin. Morphometrical analysis was performed by two different blinded operators using Image J software to obtain internal and external perimeters as well as the vessel internal (Ai) and external area (Ae) [20]. Then, internal and external radii (Ri and Re) were calculated according to the formula perimeter  = 2πR; internal diameter (lumen) was obtained by 2×Ri and medial wall thickness as Re - Ri. Media cross-sectional area (CSA) was calculated as CSA = π (Re^2^–Ri^2^). Images were obtained with a microscope (Olympus BX60) coupled to a camera (Olympus DP-72) and using 4× objective.

### Statistical analysis

Data are expressed as the mean ± SEM. One-way or two-way ANOVA followed by Bonferroni's test was performed using GraphPad Prism (GraphPad Software In). Values of p<0.05 were considered statistically significant. For two-way analysis Bonferroni's test was applied whether main factors and/or interaction were significant.

### Diet and reagents

NP and LP diets were purchased from *Pragsoluções Biociências* (Jaú, SP, Brazil) and taurine (2-aminoethanesulfonic acid) was from *Botica Ouro da Mata* Compounding Pharmacy (Campinas, SP, Brazil). MnTMPyP [Mn(III) tetrakis(1-methyl-4-pyridyl)porphyrin] pentachloride was from Calbiochem (San Diego, CA, USA). Components of Krebs-Henseleit solution, urethane, serotonin, acetylcholine, sodium nitroprusside, SOD, and apocynin were purchased from Sigma-Aldrich (Saint Louis, MO, USA). Primary antibodies: anti-eNOS from BD Transduction Laboratories (Franklin Lakes, NJ, USA); anti-phospho-eNOS (Ser 1177) and anti-CuZn-SOD from Cell Signaling Technology (Beverly, MA, USA); anti-EC-SOD and anti-Mn-SOD from Enzo Life Sciences (Farmingdale, NY, EUA); anti-p47^phox^ and anti-catalase from Sigma-Aldrich (St Louis, MO, USA); anti-gp91^phox^ from Millipore (Millipore Corporation, Bedford, MA, USA); anti-GPX and anti-α-actin from Abcam (Cambridge, MA, USA).

## Results

### Body weight, blood pressure and heart rate

There was no difference in body weight between the groups at the beginning of the study. After 90 days of diet protocol, body weight and plasma albumin content were significantly lower in rats that received the low-protein diet compared with group receiving the normal protein diet (body weight: NP = 425±15 *vs*. LP = 180±6 g, p<0.01, N = 8; albumin: NP = 3.35±0.069 *vs*. LP = 2.96±0.018 g/dL, p<0.01, N = 5). These data confirm the efficacy of the low-protein diet inducing protein restriction [16], [17]. Taurine supplementation did not alter these parameter in either group (body weight: NPT = 428±4 *vs*. LPT = 215±13 g, p>0.01, N = 8; albumin: NPT = 3.21±0.037 *vs*. LPT = 2.79±0.026 g/dL, p>0.01, N = 5) as previously demonstrated [16].

As shown in Table 1, the values of SBP, DBP and MAP were significantly higher in LP compared with NP rats. Taurine supplementation attenuated these values in LPT, but did not alter the blood pressure in NPT, compared with respective non-supplemented groups. Neither the protein-restricted diet nor taurine supplementation altered HR (Table 1).

**Table 1:** Effect of taurine (T) supplementation on blood pressure and heart rate from rats fed an isocaloric normal- (NP) or low-protein (LP) diet.

	NP (n = 7)	LP (n = 7)	NPT (n = 9)	LPT (n = 8)
SBP (mmHg)	116±3	142±2**	118±2	128±3^+#^
DBP (mmHg)	66±4	90±4**	64±4	78±4^+#^
MAP (mmHg)	84±3	110±4**	84±3	97±4^+#^
HR (bpm)	363±17	395±16	353±10	386±9

SBP: systolic blood pressure; DBP: diastolic blood pressure; MBP: mean arterial pressure; HR: heart rate. Values are mean ± SEM. The number of rats (n) is indicated in the parenthesis. 2-way ANOVA:

**p<0.001 *vs*. NP;

+ p<0.05 *vs*. LP;

# p<0.05 *vs*. NPT.

### Aortic relaxation responses, ROS generation and NO release

**Figure 1:**
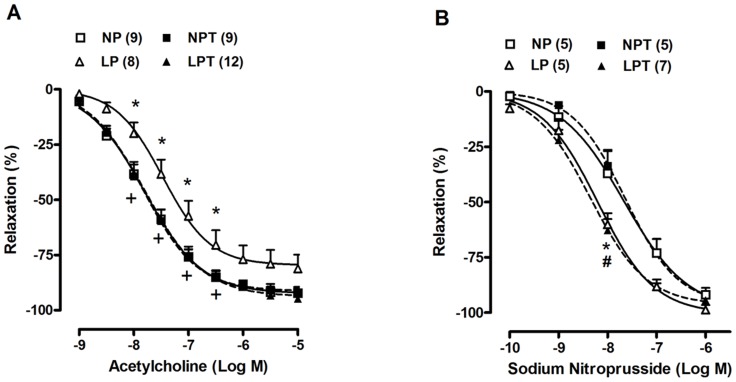
Taurine supplementation prevented the reduction in endothelium-dependent relaxation to acetylcholine induced by post-weaning protein restriction. Concentration-response curves to acetylcholine (A) and sodium nitroprusside (B) in aortas from rats fed an isocaloric normal-protein (NP) or low-protein (LP) diets without or with taurine (T). Relaxation responses are expressed as a percentage of serotonin-induced contraction. Values represent the mean ± SEM. The number of rats included in each group is indicated into parentheses in the figure. 2-way ANOVA:Figure 1A showed significant main effects of groups and acetylcholine;Figure 1B showed significant main effects of groups and sodium nitroprusside. Bonferroni post-test (p<0.05): * LP *vs*. NP, ^+^ LPT *vs*. LP, ^#^ LPT *vs*. NPT.

Relaxation responses to acetylcholine and sodium nitroprusside obtained in aortic rings are shown in Figure 1. The vasodilator response to acetylcholine was significantly reduced in aortas of LP rats compared to NP rats (Figure 1A); whereas taurine supplementation fully prevented this reduction and acetylcholine-induced relaxation of LPT was similar to NPT (Figure 1A). The relaxation response to sodium nitroprusside was enhanced in aortas of LP rats when compared to NP rats (Figure 1B). Taurine supplementation did not modify this effect (Figure 1B).

**Figure 2:**
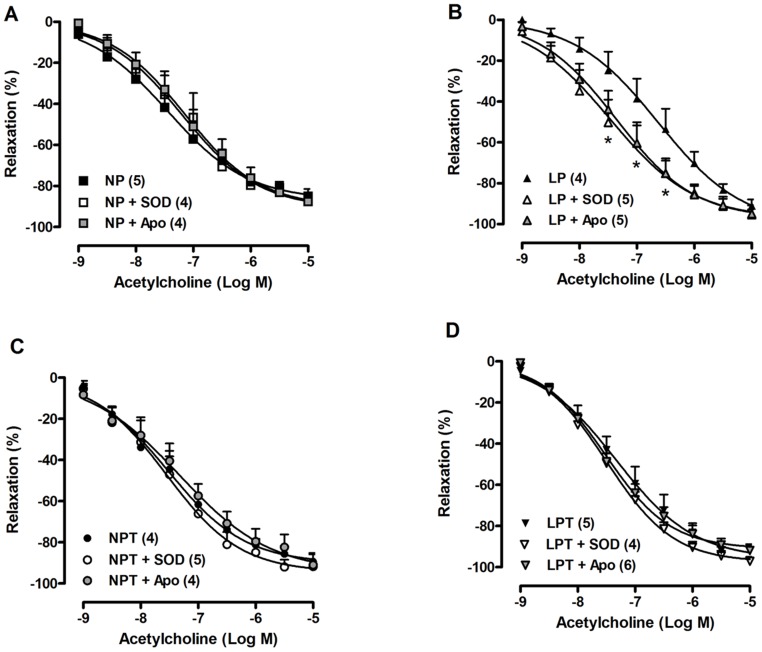
Superoxide dismutase (SOD) and apocynin (Apo) restored the endothelium-dependent relaxation in aorta from low-protein-fed rats. Concentration-response curves to acetylcholine were obtained in aortas from rats fed an isocaloric normal-protein (NP) (A) or low-protein (LP) (B) diets supplemented with taurine (T) in drinking water (C, D). Values represent the mean ± SEM. The number of rats included in each group is indicated into parenthesis in the figure. 2-way ANOVA:Figure 2B showed significant main effects of SOD/Apo and acetylcholine. Bonferroni post-test (p<0.05): * *vs*. LP.

The incubation with SOD or apocynin in aortic rings from LP rats normalized the reduced relaxation response to acetylcholine (Figure 2B); whereas neither SOD nor apocynin altered the acetylcholine-induced relaxation in NP (Figure 2A), NPT (Figure 2C) and LPT groups (Figure 2D).

**Figure 3:**
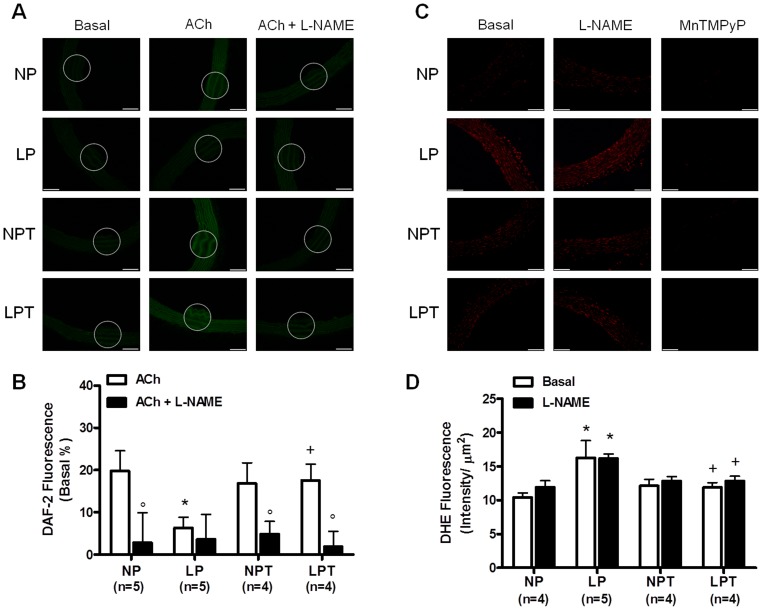
Increased ROS generation and reduced NO bioavailability induced by low-protein diet is normalized by taurine. Representative fluorographs and fluorescence intensity analysis of aortic sections labeled with NO-sensitive fluorescent dye 4,5-diaminofluorescein (DAF-2) (A, B) or with the dihydroethidine (DHE) (C, D) from rats fed an isocaloric normal-protein (NP) or low-protein (LP) diets without or with taurine (T). Fluorescence was quantified as intensity per vessel area (µm^2^); DAF-2 was evaluated in the presence of acetylcholine (ACh, 1 µM) and the results are expressed as a percentage of increase in relation to basal level. Effect of L-NAME incubation was evaluated in NO (A, B) and ROS production (C, D). Values represent the mean ± SEM. The number of rats (n) included in each group is indicated in parenthesis. 2-way ANOVA:Figure 3B showed significant main effects of groups and L-NAME;Figure 3D showed significant main effect of groups. Bonferroni post-test (p<0.05): * *vs*. NP, ^+^ *vs*. LP, ° *vs*. without L-NAME. Bar scale = 100 µm.

NO production evaluated by DAF-2 fluorescence was decreased in aortas from LP group in the presence of acetylcholine compared with the NP group (Figure 3A and 3B). Taurine treatment improved the acetylcholine-induced NO release in aortas from LPT (Figure 3A and 3B). L-NAME significantly reduced DAF-2 fluorescence in aortas from NP, NPT and LPT, but not in LP group indicating the impaired NOS-derived NO release in these animals (Figure 3A and 3B). As shown in Figure 3C and 3D, increased ROS generation was measured in aorta from LP animals (∼20%) compared with NP animals. L-NAME incubation did not change ROS production (Figure 3C and 3D) indicating no role for uncoupled eNOS. MnTMPyP completely abolished the fluorescence in response to DHE (Figure 3C) showing superoxide anion as the main ROS measured in the rat aortic sections. Taurine supplementation fully prevented the increase in superoxide anion generation induced by low protein diet (Figure 3C and 3D).

### Protein expression of NADPH subunits, antioxidant enzymes and eNOS

**Figure 4:**
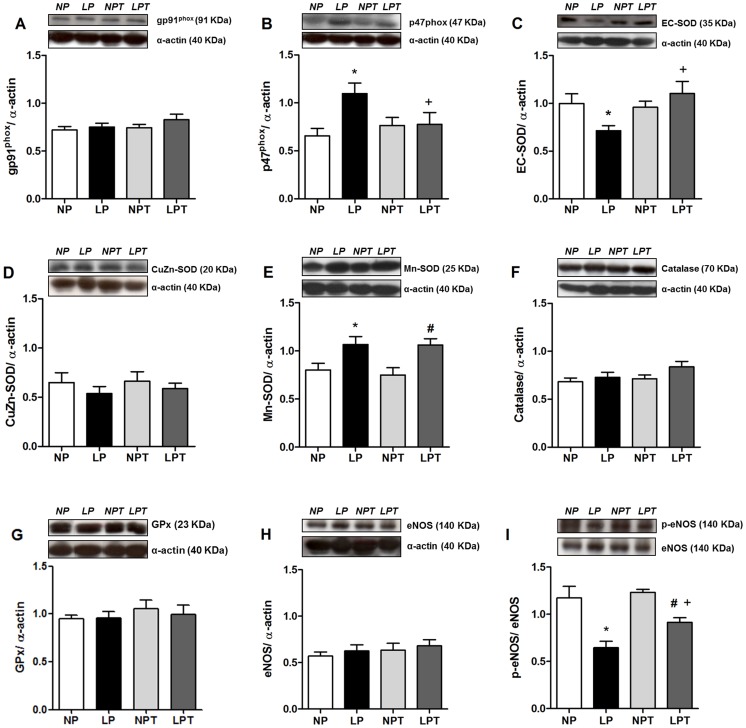
Taurine prevented the alterations in p47^phox^, EC-SOD and eNOS phosphorylation induced by low-protein diet. Protein expression of gp91^phox^ (A), p47^phox^ (B), EC-SOD (C), CuZn-SOD (D), Mn-SOD (E), catalase (F), glutathione peroxidase (GPx) (G), eNOS (H) and phosphorylated Ser1177 (p)eNOS (I) were analyzed by Western-blot in aorta from rats fed normal-protein (NP) or low-protein (LP), with or without taurine (T). α-actin content was used as internal control in each sample. Values represent the mean ± SEM (Number of animals in each group = 5–9). 2-way ANOVA:Figure 4B and 4C showed significant interaction (LP diet x taurine).Figure 4E showed main effect of LP diet;Figure 4I showed significant main effects of LP diet and taurine. Bonferroni post-test (p<0.05): * *vs*. NP, ^+^ *vs*. LP, ^#^ *vs*. NPT.

The protein expression of membrane NADPH oxidase subunit gp91^phox^ was not affected by diet or taurine (Figure 4A). However, protein expression of regulatory p47^phox^ subunit of NADPH oxidase was 75% higher in aorta from LP group compared with NP group; whereas p47^phox^ levels in LPT were similar to NPT and NP (Figure 4B). EC-SOD protein expression was 25% lower in aortas from the LP group, and this expression was rescued by taurine supplementation (Figure 4C). In contrast, Mn-SOD protein expression was increased in aortas from the LP group and taurine did not modify this effect (Figure 4E). No differences in Cu/Zn-SOD (Figure 4D), catalase (Figure 4F) or GPx (Figure 4G) expressions were observed between groups. Total eNOS expression did not differ among groups (Figure 4H); however phosphorylated eNOS at Ser 1177 was reduced in aorta from LP group and partially reversed by taurine (Figure 4I).

### Morphometric analysis

**Figure 5:**
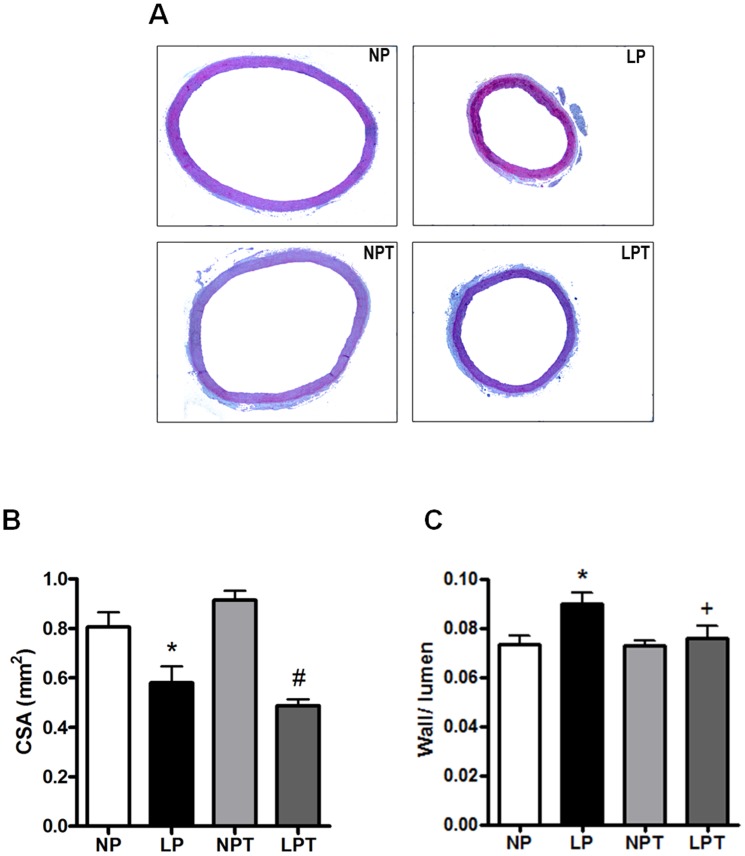
Effect of post-weaning protein restriction and taurine supplementation on the morphometric parameters of rat aorta. Representative images of transversal aortic slices are shown in panel A. Aortic cross sectional area (CSA) (B) and wall/lumen ratio (C) were calculated in aortic slices of rats fed a normal-protein (NP) or low-protein (LP) diet, with or without taurine (T). Values represent the mean ± SEM (Number of animals in each group = 5–9). 2-way ANOVA: Figure 5B showed main effect of LP diet; Figure 5C showed significant interaction (LP diet x taurine). Bonferroni post-test (p<0.05): * *vs*. NP, ^+^ *vs*. LP,^ #^ *vs*. NPT.

Morphometric analysis of aorta is shown in Figure 5. Protein restriction reduced CSA, whereas taurine supplementation did not modify this effect (Figure 5A and 5B). Lumen values were reduced in aortas from protein-restricted rats (internal diameter: NP = 1.76±0.09 *vs*. LP = 1.40±0.08 mm; p<0.05, N = 4), and this effect was not modified by taurine supplementation (NPT = 1.82±0.06 *vs*. LPT = 1.48±0.12 mm; p<0.05, N = 4). Low-protein diet did not change aortic wall thickness (NP = 135±7 *vs*. LP = 127±6 µm; p>0.05, N = 4), however taurine reduced it by 20% in LPT group (NPT = 144±4 *vs*. LPT = 112±8 µm; p<0.05, n = 4). Thus, lumen narrowing led to a higher wall/lumen ratio in LP animals that was prevented by reduction of wall thickness induced by taurine in these animals (Figure 5C).

## Discussion

In the current study, we observed that post-weaning low-protein diet increases blood pressure associated with endothelial dysfunction and vascular remodeling in adult rats. These results highlight the impact of post-weaning protein undernutrition in vascular function and structure. Furthermore, protein restriction induced vascular oxidative stress with increased ROS generation and p47^phox^ subunit protein expression; and decreased EC-SOD protein expression. Nevertheless, we are the first to demonstrate that taurine supplementation could be a beneficial intervention, preventing or ameliorating the observed cardiovascular effects of early protein restriction.

Deficiency of macronutrients during early stages of development may incur an increased risk of subsequent hypertension [21]. In the present study, low-protein-fed rats presented elevated values of systolic and diastolic arterial pressure. Previous studies have demonstrated high blood pressure in rats fed a 6% protein diet, that was associated with baroreflex dysfunction, increased sympathetic activity and reduced vagal activity to the heart [2], [8], [22], [23]. In addition, elevated blood pressure and altered endothelial function was found in rat aortic rings from rats fed low protein diet with deficiency of vitamins and minerals [24]. Herein, we demonstrated for the first that post-weaning isocaloric protein restriction is associated with endothelial dysfunction in early adulthood. These data are consistent with previous reports showing that intrauterine exposure to protein restriction results in high systolic blood pressure, endothelial dysfunction and altered vascular tone in isolated resistance arteries and thoracic aorta of the male offspring [6], [7].

The endothelial dysfunction of post-weaning protein-restricted rats was characterized by a reduced acetylcholine-induced relaxation and NO release in aorta. These effects were associated with reduced phosphorylation of eNOS at Ser1177, suggesting reduced eNOS-derived NO synthesis in vessels from low-protein-fed rats. In addition, enhanced ROS production was found in aortas from low-protein-fed rats. This oxidative stress was related to increased superoxide anion production, as the SOD mimetic MnTMPyP abolished the detection of aortic ROS. Increased superoxide anion production in vascular tissue is associated with reduced endothelium-dependent relaxation in several models of cardiovascular diseases [25], [26]. Superoxide anion could react with NO reducing its bioavailability and generating peroxynitrite [27]. In addition, it is known that superoxide anion leads to uncoupling of eNOS, reducing its activity and generating even more superoxide anion [28]. However, L-NAME incubation did not reverse the increased ROS production in aorta from LP group, indicating no role for uncoupled eNOS in this experimental model of protein restriction. Together, the results suggest besides impaired NO synthesis, the endothelial dysfunction induced by low protein diet seem to be related to increased inactivation of NO caused by excessive superoxide anion generation.

Apocynin incubation restored the vasorelaxation to acetylcholine in aortas from the low-protein-fed group. Further, the expression of the cytosolic p47^phox^ subunit of NADPH oxidase was enhanced in aortas from LP group. These results suggest that post-weaning protein restriction culminates with endothelial dysfunction related to an enhanced activity of aortic NADPH oxidase generating superoxide. In agreement, increased NADPH oxidase-derived superoxide production was associated with impaired endothelium-dependent relaxation to acetylcholine and bradykinin in mesenteric vascular bed from intrauterine-undernourished rats [29]. It is known that EC-SOD plays an important role regulating endothelial function by controlling the levels of generated extracellular superoxide anion, mostly through NADPH oxidase activity [30]. Interestingly, protein expression of EC-SOD was reduced in aorta from low-protein-fed rats, as well as SOD incubation normalized the acetylcholine-induced relaxation in this group. Thus, reduced protein levels of EC-SOD could be contributing to the endothelial dysfunction and oxidative stress induced by protein restriction. On the other hand, there was an increased content of Mn-SOD in the aorta of animals subjected to protein restriction. Mn-SOD (or SOD2) is the mitochondrial isoform of SOD that catalyzes dismutation of superoxide anion generated by respiratory chain activity; this isoform can be induced by various oxidizing agents to protect against oxidant insults [31]. Thus, an increased mitochondrial ROS generation in low-protein-fed rats could be leading to increased Mn-SOD expression, but it did not prevent oxidative stress.

Despite reduced relaxation to acetylcholine and oxidative stress, the vasodilation induced by sodium nitroprusside, a NO donor, was enhanced in aortas from protein-restricted rats. It is known that the downstream cascade of NO could be upregulated in cardiovascular diseases associated with reduced synthesis and/or bioavailability of NO, despite enhanced levels of vascular superoxide anion [25]. It suggests a vascular compensatory mechanism to the endothelial dysfunction and oxidative stress induced by protein restriction.

Taurine has emerged as a potential therapeutic target for cardiovascular diseases, preventing hypertension, stroke and atherosclerotic arterial diseases [32]. We observed here that 2.5% taurine supplementation has an antihypertensive effect in adult rats subjected to post-weaning protein restriction. Efficiency of the taurine supplementation protocol used in the present study was previously described: it increased plasma taurine by 2.4-fold in NPT and 3.4-fold in LPT rats compared with respective non-taurine treated groups, and without differences between NPT and LPT taurine levels [16]. Consistent with the present results, 2.5% taurine supplementation reduced blood pressure values of gestational protein-restricted offspring in mice [33], as well as it showed protective vascular effect in aorta from rabbit fed high cholesterol diet [34]. Interestingly, deficiency of taurine is found in patients with essential hypertension [35] and an antihypertensive effect of taurine was reported in patients with borderline hypertension [36]. Therefore, our present work reproduced previous findings in hypertensive humans and extends the knowledge by suggesting a beneficial role of this amino acid in the vascular alterations induced by low protein diet.

Taurine supplementation restored the endothelial function in young smokers [37], as well as in type I diabetic rats [38], [39]. Here, chronic taurine supplementation restored the impaired endothelium-dependent acetylcholine-induced relaxation in aorta from protein-restricted rats, associated with reduced ROS generation and enhanced acetylcholine-induced NO release. Three main mechanisms seem to be related to this protective endothelial effect of taurine supplementation in low-protein-fed rats: 1) reduced vascular NADPH oxidase-derived superoxide anion production, with normalization of the expression of the cytosolic subunit p47^phox^; 2) partial restoration of phosphorylation of eNOS at Ser1177 and; 3) enhanced expression of EC-SOD.

Previous studies had attempted to connect the antioxidant effect of taurine ameliorate endothelial function. It was shown that taurine supplementation improved the diabetes-induced endothelial dysfunction of rat corpus cavernosum associated with a reduction in the protein expression of gp91^phox^ indicating a protective role of taurine inhibiting endothelial NADPH oxidase activity [40]. In addition, a previous study has demonstrated that taurine can act as antioxidant by preventing the reduction of the expression and secretion of EC-SOD in cultured vascular smooth muscle cells isolated from rat thoracic aorta, in accordance with the results from present study [41].

Finally, protein restriction induced an inward hypotrophic remodeling of aorta, with reduction of vascular lumen and CSA. This hypotrophic remodeling might be associated with low total protein content [16] or a feedback mechanism to counteract endothelial dysfunction [42]. In agreement, a reduced smooth muscle area was reported in mesenteric resistance arteries of offspring of low protein-fed females [42]. The reduction in CSA was not reversed by taurine, suggesting no changes in the hypotrophic effect of protein restriction in vascular smooth muscle. This observation is in accordance with no effect of taurine on total protein content or on body mass of low-protein-fed rats [16]. However, taurine prevented the reduction in wall/lumen ratio through a reduction in media thickness. We could not exclude that this improvement of aortic remodeling induced by taurine is mediated via its effect lowering blood pressure. Together, the data suggest an important role of taurine preventing vascular remodeling and hypertension occurring in a metabolic disorder.

In summary, this study shows for the first time that post-weaning protein restriction disrupts redox homeostasis related to a reduced vascular expression of EC-SOD and increased expression of the p47^phox^ subunit of NADPH oxidase. These are most likely mechanisms enhancing ROS generation, inducing endothelial dysfunction and vascular remodeling caused by protein restriction. Taurine supplementation attenuated the increase in blood pressure and prevented vascular alterations induced by low-protein diet. Therefore, the present data put forward the beneficial effects of taurine supplementation on the cardiovascular damage induced by undernutrition in early life.
